# Cytoplasmic Plaque Formation in Hemidesmosome Development Is Dependent on SoxF Transcription Factor Function

**DOI:** 10.1371/journal.pone.0043857

**Published:** 2012-09-04

**Authors:** Shelly Oommen, Mathias Francois, Maiko Kawasaki, Melanie Murrell, Katsushige Kawasaki, Thantrira Porntaveetus, Sarah Ghafoor, Neville J. Young, Yoshimasa Okamatsu, John McGrath, Peter Koopman, Paul T. Sharpe, Atsushi Ohazama

**Affiliations:** 1 Craniofacial Development and Stem Cell Biology, and Biomedical Research Centre, Dental Institute, King's College London, London, United Kingdom; 2 Institute for Molecular Bioscience, The University of Queensland, Brisbane, Australia; 3 Department of Periodontology, Showa University Dental School, Tokyo, Japan; 4 Genetic Skin Disease Group, St John's Institute of Dermatology, Division of Skin Sciences, King's College London, London, United Kingdom; University of Colorado, Boulder, United States of America

## Abstract

Hemidesmosomes are composed of intricate networks of proteins, that are an essential attachment apparatus for the integrity of epithelial tissue. Disruption leads to blistering diseases such as epidermolysis bullosa. Members of the *Sox* gene family show dynamic and diverse expression patterns during development and mutation analyses in humans and mice provide evidence that they play a remarkable variety of roles in development and human disease. Previous studies have established that the mouse mutant ragged-opossum (*Ra^op^*) expresses a dominant-negative form of the SOX18 transcription factor that interferes with the function of wild type SOX18 and of the related SOXF-subgroup proteins SOX7 and −17. Here we show that skin and oral mucosa in homozygous *Ra^op^* mice display extensive detachment of epithelium from the underlying mesenchymal tissue, caused by tearing of epithelial cells just above the plasma membrane due to hemidesmosome disruption. In addition, several hemidesmosome proteins expression were found to be dysregulated in the *Ra^op^* mice. Our data suggest that SOXF transcription factors play a role in regulating formation of cytoplasmic plaque protein assembly, and that disrupted SOXF function results in epidermolysis bullosa-like skin phenotypes.

## Introduction

**Figure 1:**
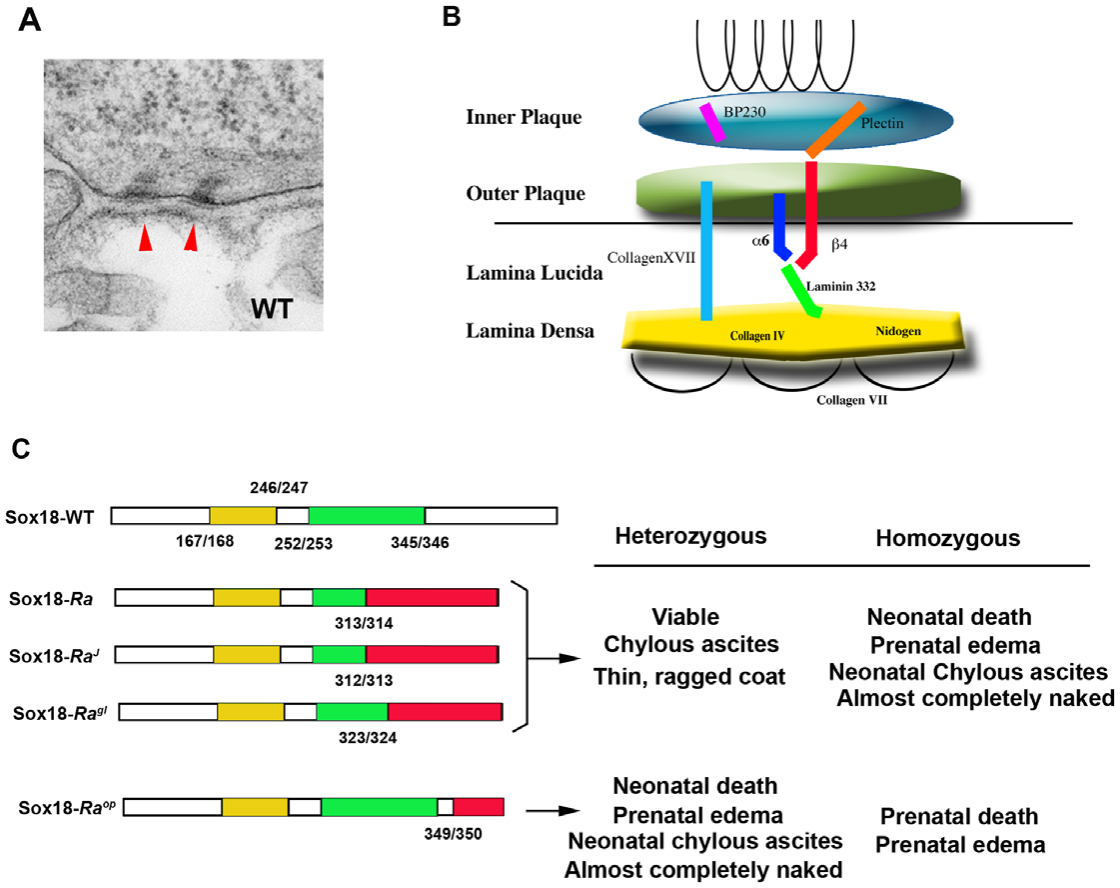
Components in hemidesmosome and Sox18 protein in *Ra* mice. TEM image of wild-type hemidesmosomes in the oral cavity (arrows in A). Schematic representation of molecular organization of basement membrane zone including hemidesmosomes (B). (C) Schematic representation of the Sox18 proteins in *Ra* mice. The numbering indicates the amino acid coordiates of the represented boxes.

Epithelial tissue integrity is a critical feature of organ formation and function that is maintained through several types of cell junction including hemidesmosomes, desmosomes, gap junctions and tight junctions. All these junctions are composed of intricate networks of proteins. Hemidesmosomes are rivet-like structures present on the inner aspect of the basal plasma membrane (Fig. 1A). These junctions constitute the main adhesion units of the basement membrane zone, which contribute to the attachment of epithelial cells to the underlying basement membrane. Hemidesmosomes are composed of an electron-dense inner plaque into which intermediate filaments are inserted, and an outer plaque that lies on the plasma membrane. An electron-dense region, parallel to the plasma membrane, called the lamina densa and an electron-lucent zone called the lamina lucida are identified subjacent to the basal epithelium. Anchoring filaments traverse the lamina lucida space [1]–[3].

Hemidesmosomes were once thought to be halves of desmosomes that form adherens junctions between epithelial cells. However, hemidesmosomes are believed to be composed mostly of different molecules from desmosomes [4]. Hemidesmosomes do not contain desmoplakin and desmoglein that are found in desmosomes, but contain their own specific molecules such as BPAG1e (BP230). The hemidesmosome-basement membrane complex contains many proteins including plectin, BPAG1e and less well-characterized proteins that are part of the cytoplasmic plaque proteins (Fig. 1B). BP180 (collagen XVII, BPAG2) and integrin α6ß4 are hemidesmosomal transmembrane molecules, laminin 332 is an anchoring filament and collagen IV and nidogen are located in the lamina densa.

Inherited mutations of hemidesmosome proteins or acquired autoantibodies against hemidesmosome molecules result in blistering diseases such as epidermolysis bullosa and bullos pemphigoid, respectively. To date, several molecules including keratin 5 and 14, plectin, collagen XVII, laminin 332 and integrin α6ß4 have been identified as being mutated in epidermolysis bullosa [5]–[7]. In addition to epithelial integrity, hemidesmosomes also play a critical role in cell migration, cell-stromal coherence, polarization, spatial organization, tissue architecture, wound healing and tissue morphogenesis [1], [8]–[11].

Members of the SOX (Sry-type HMG box) gene family encode transcription factors that show dynamic and diverse expression patterns during development. Analysis of mutations in humans and mice suggest that they play multiple roles during development [12]–[14]. The mouse mutation *ragged* is semi-dominant and arose spontaneously in a crossbred stock of mice [15]. *Ragged* mice are characterized by abnormalities in their coat and cardiovascular system. Recently, *Sox18* was identified as the mutated gene responsible for the *ragged* phenotype [16]. Point mutations were found in *Sox18* in *ragged* mice that result in missense translation and premature truncation of the encoded protein producing a dominant negative transcription factor that suppresses the endogenous function not only of wild type SOX18 but also of the highly related SOXF-subgroup proteins SOX7 and SOX17. Three other *ragged* alleles, *ragged-like*, *ragged-J* and *opossum* (*Ra^op^*) have also been reported [17] with a similar phenotype but with variable severity. Heterozygotes are viable and healthy with thin, ragged coats compromised of guard hairs and awls, but lacking auchenes and zigzags (Fig. 1C) [15]. Homozygotes lack vibrissae and coat hairs, display oedema and rarely survive past weaning depending on the genetic background. Unlike the other three alleles, *Ra^op^* represents a more severely affected class of mutant, with heterozygotes resembling homozygotes of the other three alleles (Fig. 1C) [17].

Mouse *Sox18* encodes a 468 amino acid protein with an N-terminal domain of unknown function, a 79 amino acid HMG domain shown to bind the consensus SOX binding sequence AACAAAG, a 93 amino acid transcriptional *trans*-activation domain, and a 123 amino acid C-terminal domain that is highly conserved between species. *Ra^op^* mice showed a single base deletion resulting in a C-terminal translation frameshift and premature termination of SOX18 at 435 amino acids. The HMG domain is intact in all four alleles of *ragged* mice, but the mutant proteins are unable to activate transcription, explaining their dominant-negative action.

Targeted inactivation of *Sox18* by deleting the HMG domain resulted in no obvious cardiovascular defects and only a mild coat defect with a reduced proportion of zigzag hairs [18] in a mixed genetic background; the mild phenotype suggests compensation by SOX7 and/or SOX17. By contrast, on a C57/Bl6 genetic background, knockout mouse embryos die *in utero* at E14 due to a massive generalized oedema [19]; SOX7 and −17 are therefore able to compensate for the loss of SOX18 in some tissues but not others, and only in certain strains of mice [20]. Therefore, *Ra^op^* mice represent a valuable tool for studying SOXF function, since the functions of all three SOXF factors are essentially ablated at once.

We show here that homozygous *Ra^op^* mice display extensive detachment of epithelium in skin and oral mucosa. Integrin ß4 and α6, and plectin proteins are affected in the homozygous *Ra^op^* mice. SOXF transcription factors are therefore essential for hemidesmosome formation. One or more SOXF transcription factors may therefore be linked to epidermolysis bullosa variants associated with abnormal hemidesmosome attachment complexes.

## Results and Discussion

### Expression of *Sox18* and related *SoxF* genes

**Figure 2:**
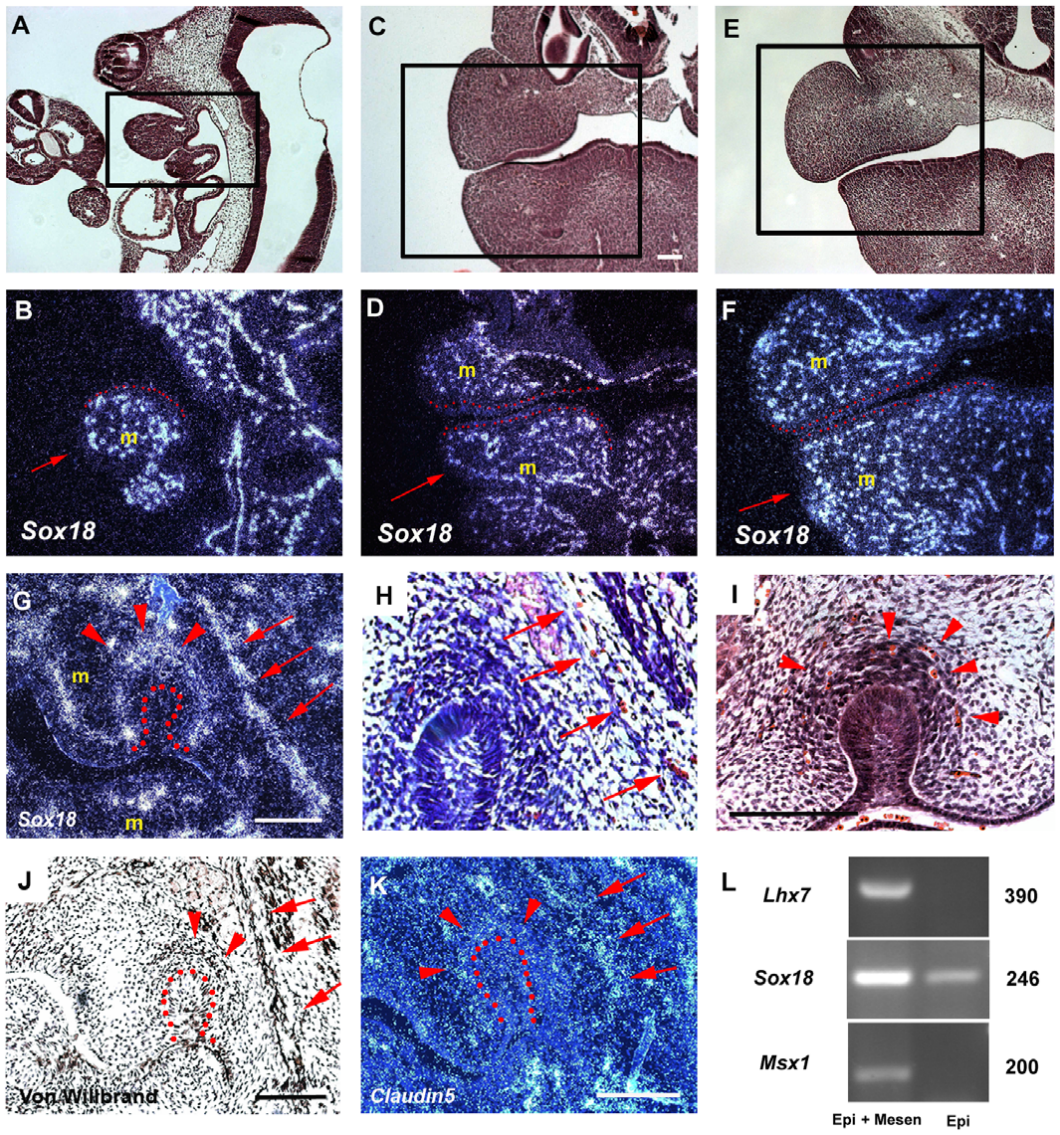
*Sox18* expression in head. Radioactive *in situ* hybridisation showed strong expression of *Sox18* in mesenchyme (m) at E9.5 (B), E10.5 (D), E11.5 (F) and E13.5 (G) of wild-type. Insets of A, C and E represent histological images of B, D and F, respectively. Arrows indicate mandible (B–F). A, B; Sagittal section, C–K; Frontal sections. B, D, F; Epithelia of jaws outlined by red dots. (G) *Sox18* expression is observed outside of condensed mesenchyme of tooth germs (arrowheads) and the buccal regions of maxillary jaws (arrows). (H, I) Blood cells were observed at the buccal side of maxillary mesenchyme (arrows in H) and outside the condensed mesenchyme of tooth germs (arrowheads in I). von-Willbrand factor proteins were detected in the same regions (arrowheads and arrows in J). The endothelial marker gene, *Claudin 5* showed a similar expression pattern to *Sox18* (arrowheads and arrows in K). (G, J, K) Tooth germ epithelium is outlined by red dots. L; RT-PCR analysis showed the presence of *Sox18* expression and absence of *Msx1* or *Lhx7* in epithelium. Epi, total RNA extracted from only epithelium of mandible; Epi+Mesen, total RNA extracted from whole mandibles. Scale bars: 100 µm (C–F); 125 µm (G, I–K).

The expression of *Sox18* was analyzed in the developing jaws of mouse embryos between days 9.5 and 13.5 of gestation (E9.5–E13.5) using radioactive *in situ* hybridization. *Sox18* was expressed strongly in mesenchyme at all stages (Fig. 2). A punctate expression pattern of *Sox18* was seen throughout the mesenchyme at E9.5–E12.5 (Fig. 2B, 2D, 2F). At E13.5, *Sox18* expression was restricted to the outer cells of condensed tooth mesenchyme and the buccal region of the maxilla (Fig. 2G). The distribution of *Sox18* mRNA was consistent with expression in endothelial cells, which was confirmed by the expression of Von Willbrand factor protein (Fig. 2G–2J). *Claudin5* is an endothelial cell-specific protein and its expression pattern was found to be identical to *Sox18* expression in the mesenchyme at E13.5, consistent with reports that *Sox18* is involved in endothelial cell differentiation (Fig. 2K) [16], [17], [21]–[23].

In addition to expression in the mesenchyme, faint expression of *Sox18* was also found in the oral epithelium at these stages. In order to confirm this expression, RT-PCR analysis was performed using total RNA extracted from oral epithelium or from whole mandibles at E11.5. PCR products of the expected size were clearly detected for *Sox18* in both RNA samples (Fig. 2L). To determine if mesenchymal cells contaminated the epithelial sample, mesenchymal marker genes (*Msx1* and *Lhx7*) [24], [25] were also examined by RT-PCR using the same RNA samples. Neither *Msx1* or *Lhx7* could be detected in RNA from the epithelium whereas both genes were found in RNA from whole mandibles. The results of the *in situ* hybridization and RT-PCR expression analysis showed that *Sox18* is weakly expressed in the oral epithelium.

**Figure 3:**
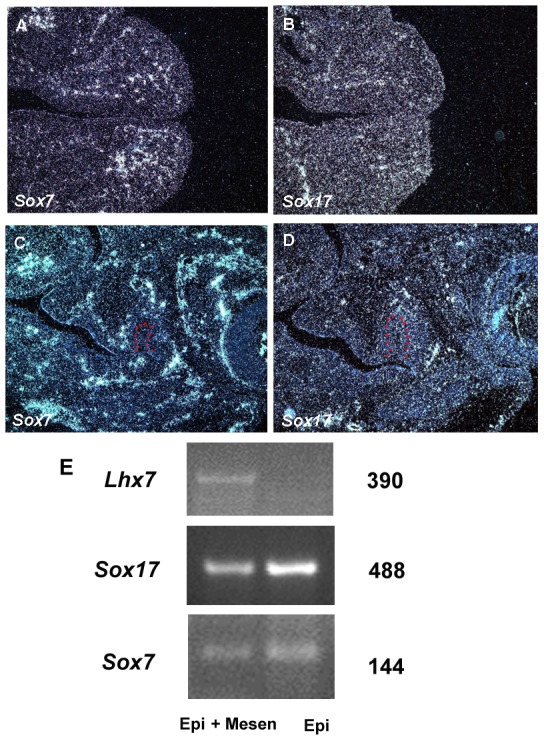
*Sox7* and *Sox17* expression in developing heads. Expression of *Sox7* (A, C) and *Sox17* (B, D) in heads at E11.5 (A, B) and E12.5 (C, D). Radioactive *in situ* hybridisation on wild type frontal sections. Tooth germ epithelium is outlined in red. E; RT-PCR analysis showed the presence of *Sox17* and *Sox7* expression and absence of *Msx1* or *Lhx7* in epithelium. Epi, total RNA extracted from only epithelium of mandible; Epi+Mesen, total RNA extracted from whole mandibles.

Since it is known that *Sox7* and/or *Sox17* can act redundantly with *Sox18* during vascular development [26], [27], we also assessed whether these two genes are expressed in the oral mucosa. We observed that both *Sox7* and *Sox17* were weakly expressed in the oral epithelium, and also showed comparable level of expression to *Sox18* in the mesenchyme (Fig. 3).

### Epithelial phenotype in *Ra^op^* mice

**Figure 4:**
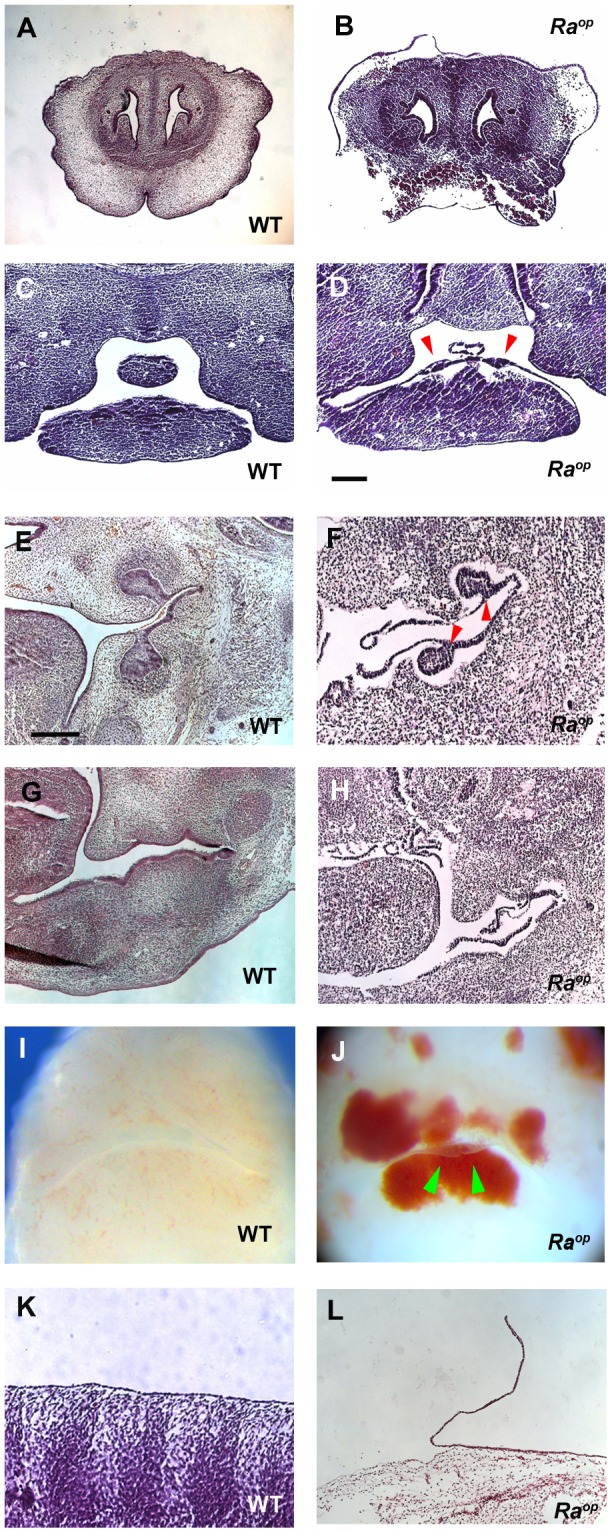
The epithelial phenotype of *Ra^op^* mice. Normal attached epithelium was observed in wild-type head (A, C, E, G). By contrast detached epithelium was observed in the skin of the snout (B), the mucosa of the incisor region (D), molar region (F), diastema (H) and the skin of trunk (L) of *Ra^op^* mice. (D, F) Arrowheads indicate tooth germs. E12.5 (A–D, I, J) and E14.5 (E–H, K, L). Detachment of epithelium was found before histological processing (green arrowheads in J). Scale bars: 125 µm (C, D); 300 µm (E–H).

To further study the potential roles of SOXF transcription factors in this system, we studied jaw development in homozygous *Ra^op^* embryos in which the functions of all three SOXF factors was expected to be suppressed, eliminating the possibility of genetic redundancy. At E12.5, detachment of the epithelium was observed in the oral mucosa and skin in *Ra^op^* mouse heads (Fig. 3). The detached epithelium retained its continuity with the oral mucosa with small areas remaining attached. Detached epithelium was observed before any processing for histology, suggesting that the detachment of epithelium in *Ra^op^* mice was not an artifact (Fig. 4J). The separation of epithelium was a fully penetrant phenotype in the oral mucosa, although the severity of the detachment was variable between individual *Ra^op^* mice examined. Detachment of the epithelium was also observed in the skin of the trunk (Fig. 4L). Heterozygotes *Ra^op^* mice showed no detachment of epithelium (Fig. S1). The less exposed surfaces of the embryonic oral cavity offered some protection to complete loss of epithelium as observed in the skin and we thus concentrated our subsequent analysis on the oral mucosa.

**Figure 5:**
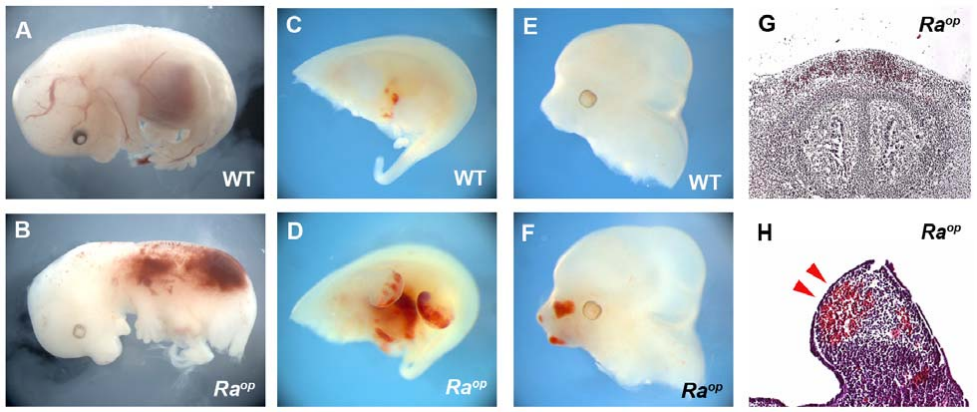
Haemorrhage in *Ra^op^* mice. Mass of blood seen in the trunk (B), limb bud (D, H) and head (F, G) of *Ra^op^* mice. (A, C, E) wild type mice. Detached epithelium could not be detected in the limb bud where mass of blood was observed (arrowheads in H). E12.5 (C–H) and E14.5 (A, B).

It has been shown that *Ra^op^* mice suffer from edema due to lymphatic vascular defects [16], [17], [19]. The mass of blood was observed in the head and trunk, including limb buds of *Ra^op^* mice. These regions of edema did not show evidence of significant epithelial detachment, suggesting that the detachment was not caused by only extravasated fluids (Fig. 5).

### Hemidesmosomes in *Ra^op^* mice

In wild-type embryos at E13.5 the tooth epithelium invaginates into the underlying mesenchyme to form epithelial buds. In *Ra^op^* mice, epithelial tooth buds were seen but the bud epithelium was detached from the mesenchyme (Fig. 4D, 4F). No obvious tears in the epithelium could be seen, suggesting that the detachment was occurring at the basement membrane.

**Figure 6:**
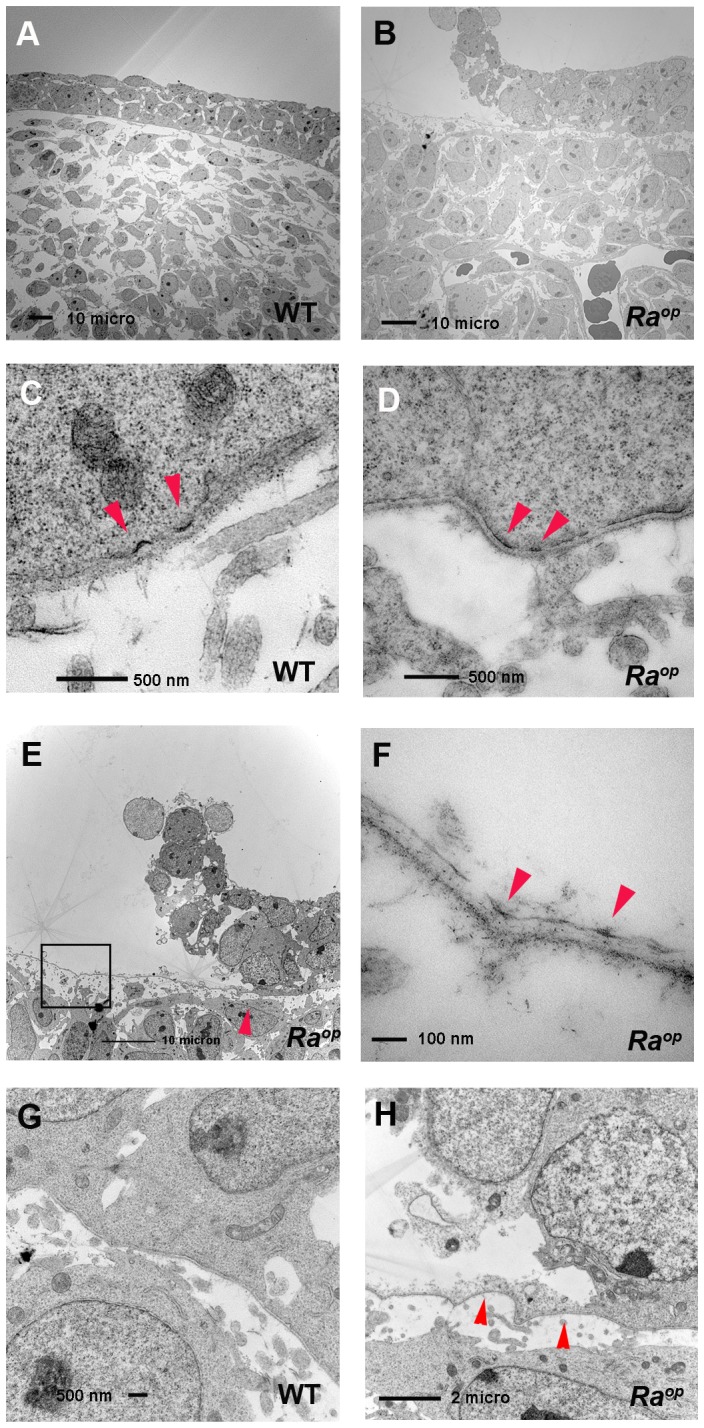
Ultrastructural analysis of *Ra^op^* mice. Frontal sections of wild-type (A, C, G) and *Ra^op^* mice (B, D, E, F, H) at E12.5. Epithelium consisted of two or three epithelial cell layers in unaffected regions of *Ra^op^* mice (B). (C) Cytoplasmic plaques were seen as single plaque structures in wild-type (arrowheads). Cytoplasmic plaques were seen as single plaques in unaffected regions of *Ra^op^* mice (D). (E, F, H) Detached epithelium of *Ra^op^* mice. (F) High magnification images taken from boxed regions in panel E. Lamina densa and lamina lucida were retained on the mesenchymal side (arrowheads in F). (H) High magnification of region indicated by arrowhead in E. Cytoplasmic plaque were also seen and retained at basement membranes of affected region of *Ra^op^* mice (arrowheads in H).

We therefore investigated the ultrastructure of the basement membrane zone in *Ra^op^* mice by transmission electron microscope (TEM) analysis. At E12.5 the epithelium of the oral mucosa of both wild-type and *Ra^op^* mice consists of two or three cell layers (Fig. 6A, 6B). Hemidesmosomes are seen as a single plaque structure at E12.5 in wild-type mice (Fig. 6C) [28]. Most of these structures were still observed as single plaques at E14.5. In *Ra^op^* mice, developing hemidesmosomes were found as single plaques in undetached regions of epithelium at E12.5 (Fig. 6D). Unaffected regions of epithelium in *Ra^op^* mice looked similar to wild-type epithelium, but in detaching areas epithelial cells were found to be torn. The splits occurred through the region of the inner plaques or close to where intermediate filaments insert into hemidesmosomes, since the lamina densa, plasma membrane and outer hemidesmosomal plaques were seen at the mesenchymal side (Fig. 6E, 6F). Nevertheless, no significant reduction in the number of hemidesmosomes was found in *Ra^op^* mice. The weakest attachment apparatus in the basement membrane zone has been thought to be the lamina lucida. Previous papers have hypothesized that the lamina lucida may be an artifact created when tissue is processing [29]. Therefore the detachment should occur in the lamina lucida, with separation being caused by mechanical stress. However, the lamina lucida was found to be intact in *Ra^op^* mice, suggesting that the increased in fluid through edema formation is unlikely to lead to the separation. On the other hand, we cannot exclude the possiblity that the extravasated fluid tear of the cytoplasm of undetached epithelial cells, since some epithelial cells did show a little retained cytoplasm at the mesenchymal side, whereas such changes were never observed in wild-type cells (Fig. 6H, 6G).

**Figure 7:**
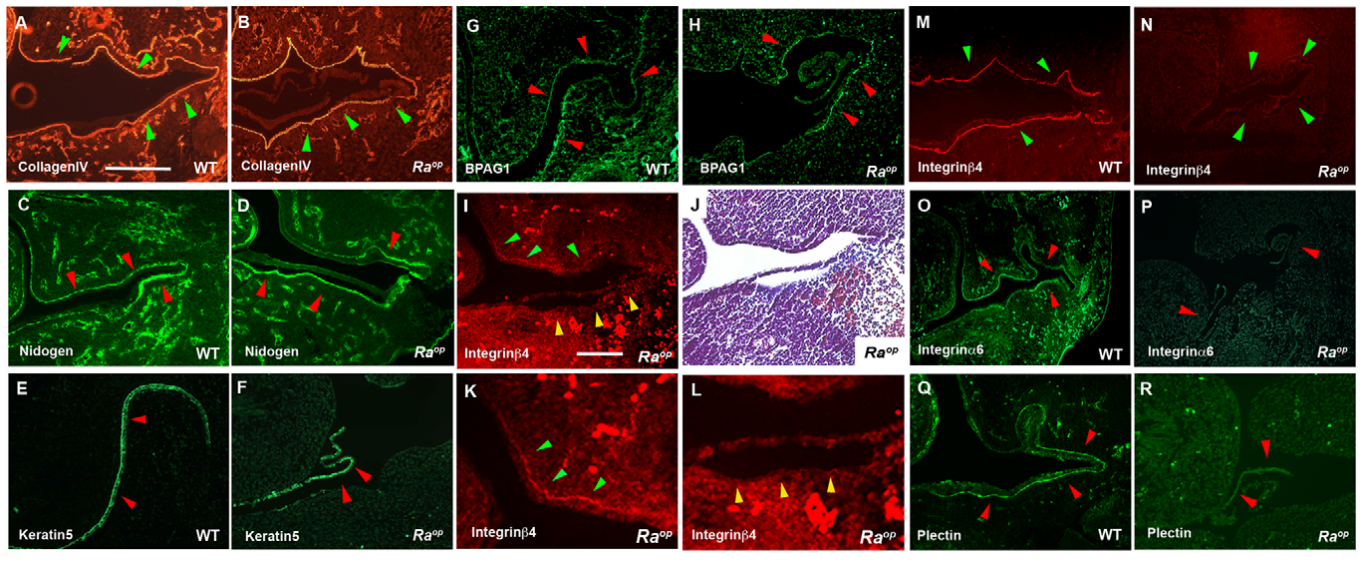
Hemidesmosome proteins of *Ra^op^* mice. Immunohistochemistry showed that there was no significant changes in Collagen IV (green arrowheads in B), nidogen (red arrowheads in D), Keratin 5 (red arrowheads in F) and BPAG1 (red arrowheads in H) where epithelium was detached in *Ra^op^* mice. Collagen IV, nidogen and BPAG1 were retained at mesenchyme, while Keratin 5 was observed in detached epithelium. (J) E12.5 mutant embryos showed both detached and undetached epithelium. (I, K, L) At E12.5 integrin ß4 level was decreased in intensity where epithelium was unaffected (green arrowheads), whereas complete lack of integrin ß4 protein was observed in detached epithelium (yellow arrowheads). J; adjacent section to I. K and L; High magnification of unaffected (K) and affected (L) region of I. At E14.5, integrin ß4 (green arrowheads in L), integrin α6 (red arrowheads in N) and plectin (red arrowheads in P) were not observed in *Ra^op^* mice. WT; A, C, E, G, M, O, Q. *Ra^op^* mice; B, D, F, H–L, N, P, R. Scale bars: 300 µm (A–H, M–R); 125 µm (I–L).

**Figure 8:**
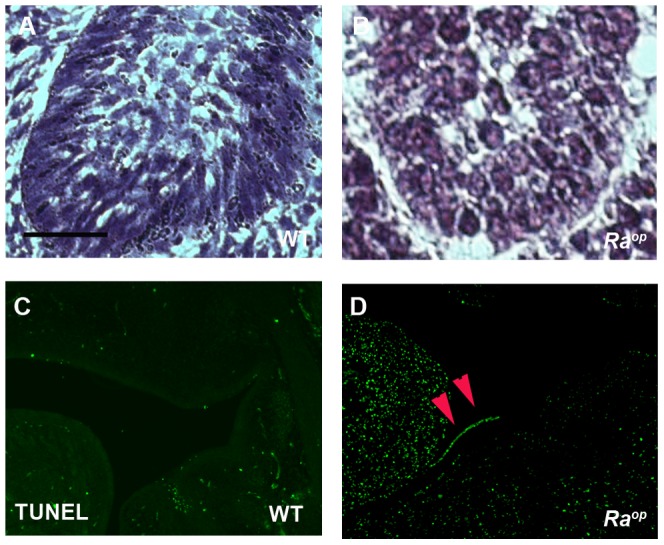
Apoptotic cells in *Ra^op^* mice. Pearl-like organization of some groups of cells in the basal tooth epithelium of *Ra^op^* (B) contrast with the normal organization observed in wild-type. Apoptotic cells were seen in epithelium of *Ra^op^* mice (arrowheads in D) but were absent in wild-type embryos. (C, D) TUNEL assay. Scale bars: 30 µm (A, B).

To assess whether the *Sox18* mutation affected the expression or localization of hemidesmosome proteins, immunohistochemistry was performed. It is known that the components of the lamina densa; collagen IV and nidogen, play a critical role in attachment of the epithelium to underlying tissues [1]–[3]. Both collagen IV and nidogen were retained on the surface of the mesenchyme where the epithelium detached in *Ra^op^* mice (Fig. 7B, 7D). These findings confirmed a lack of lamina densa disruption in *Ra^op^* mice. Keratin 5 expression was preserved in *Ra^op^* mice, mapping to the roof of the split, suggesting that intermediate filaments were unlikely to be affected (Fig. 7F). It has been reported that detachment of the epithelium is also observed in *integrin ß4-null* mice [30]–[32]. At E12.5 integrin ß4 was present but reduced at sites where the epithelium was intact in *Ra^op^* mice. However, expression of integrin-ß4 was completely abolished where the epithelium was detached in *Ra^op^* mice (Fig. 7I–7L). By E14.5 integrin-ß4 was found to be absent from all oral epithelium in *Ra^op^* mice (Fig. 7N) when compared to the wild-type mouse (Fig. 6M). A reduction in integrin α6 and plectin, has also been reported in *integrin ß4-null* mice [30], [31]. In common with *integrin-ß4-null* mice, integrin α6 and plectin was also absent in *Ra^op^* mice at E14.5 whereas their strong expression could be observed in wild-type (Fig. 7O–7R). Interaction between integrin ß4 and plectin is involved in the hemidesmosome formation, since *plectin* mutant mice also show detached epithelium in the oral mucosa, and integrin-ß4 is substantially reduced in *plectin-null* mice [33]. Interestingly, pearl-like epithelial cells were found in *integrin ß4-null* mice and were also observed in the epithelium of *Ra^op^* mice but not in wild-type (Fig. 8A, 8B) [31].

Integrin ß4 is implicated in cell survival and cell-cycle control in epithelium, since apoptotic cells are found at sites of detached epithelium in the *integrin ß4-null* mice [32]. TUNEL analysis revealed the presence of apoptotic cells in detached epithelium of *Ra^op^* mice, whereas no apoptosis could be detected in wild-type animals (Fig. 8C, 8D). BPAG1 is retained in both *integrin ß4-null* and *plectin-null* mice, and also in *Ra^op^* mice (Fig. 7H) [30], [31], [33]. Appearance of the pearl-like epithelial cells and apoptotic epithelial cells, and retention of BPAG1 in *Ra^op^* mice is thus consistent with those in *integrin ß4-null* mice. Mice with targeted deletion of other epithelial integrins α3, α6 and *ß*1 also show extensive blistering of the skin and mucous membranes [32], [34]–[36]. However, integrin ß4 is retained in these mutants, suggesting that SOXF factors are involved in hemidesmosome formation through direct or indirect modulation of either integrin ß4 or plectin or both.

**Figure 9:**
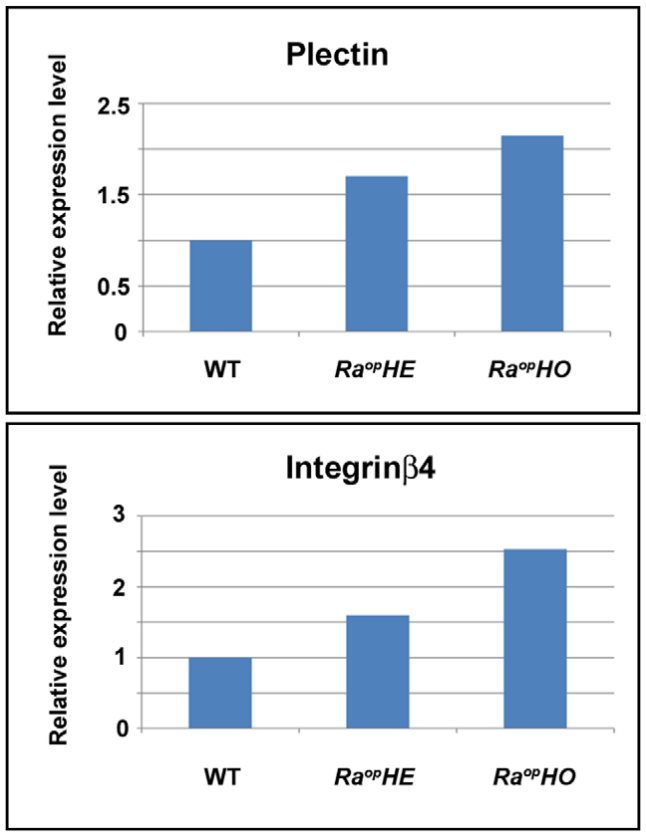
mRNA level in *Ra^op^* mice. Quantitative PCR analysis of Plectin (upper) and integrin β4 (lower) expression level. *Ra^op^HE*; heterozygous *Ra^op^* mice. *Ra^op^HO*; homozygous *Ra^op^* mice.

Cytoplasmic plaques are preserved but a reduced number of hemidesmosomes are seen in *plectin-null* mice [33]. On the other hand, cytoplasmic plaques are not detected in *integrin- ß4* mutants [30], [31], [33]. Unlike *integrin ß4-null* or *plectin-null* mice, cytoplasmic plaques were preserved and hemidesmosome number was normal in *Ra^op^* mice. Since *plectin-null* mice also exhibit muscular dystrophy, and *integrin ß4* and *plectin* mutation are associated with pyloric atresia which are not observed in *Ra^op^* mice, SOXF factors are unlikely to be direct regulator for *integrin ß4* or *plectin* [31], [33], [37], [38]. However further validation of dysregulation of these molecules was observed at the mRNA level by qPCR in whole head analysis (Fig. 9). SOXF is thus involved in intricate mechanisms in hemidesmosome formation.

Epidermolysis bullosa is a heterogeneous group of rare inherited skin and mucous membrane disorders and is divided into three major types based on the morphological level of blister formation. In addition to the results of ultrastructural analysis, BPAG1, nidogen and collagen IV were retained on the surface of the mesenchyme where epithelium was separated in *Ra^op^* mice (Fig. 7B, 7D, 7H) whereas Keratin 5 was found in the detached epithelium (Fig. 7F). These observations suggest that the blisters of *Ra^op^* mice may be classified as epidermolysis bullosa simplex that shows blisters within the epithelial cells. Epidermolysis bullosa simplex is also divided into several subclasses. Mutation of *Keratin5* or *14* have been found in most cases of epidermolysis bullosa simplex, although some epidermolysis bullosa simplex also show the mutations of *Plectin*. However, there are other occurrences of the disease that show no mutation with these genes [1]. This suggests that other genes or regulators of gene expression may account for these unresolved cases. It is also known that complete ablation of *collagen XVII* or *integrin α6ß4* results in junctional epidermolysis bullosa that shows blisters within the lamina lucida. However, it has been reported that abnormalities in these transmembrane proteins additionally leads to intracellular separation consistent with epidermolysis bullosa simplex [39], [40]. The mechanisms of the phenotype remain unknown. It is conceivable that SOXF factors are involved in these cases.

During wound healing, tumorigenesis and cell migration, cells are known to lose hemidesmosomes [41]–[43]. It has been shown that proteolytic processing and cleavage of hemidesmosome proteins are involved in the loss of hemidesmosomes [44]–[49]. We thus cannot exclude the possibility that hemidesmosome phenotypes in *Ra^op^* mice are caused by ectopic protease activity, rather than the failure of hemidesmosome protein formation.

### SOXF factors and the aetiology of epidermolysis bullosa

Mutations in *Sox18* underlie recessive and dominant forms of hypotrichosis-lymphedema-telangiectasia in humans [50]. However blistering has not been reported in for this human syndrome. Only one family suffering from hypotrichosis-lymphedema-telangiectasia, showed a mutation that corresponded to the same position as the murine *ragged* mutation. These patients were heterozygous for the mutant allele and no homozygous patients have been identified to date.

Our present findings establish that defective function of SOXF transcription factors in dominant negative *Sox18*-mutant mouse embryos recapitulates features of the human disorder epidermolysis bullosa. We also report that homozygous *Ra^op^* embryos are characterized by a failure to assemble hemidesmosomes in the oral epithelium. This lack of hemidesmosome formation correlates with the lack of expression of key components of the inner plaque (plectin) and the outer plaque (integrin ß4). Our data suggest that SOX18 and/or its related SOXF transctiption factors SOX7 and −17 may play a critical role in modulating the expression of genes involved in cell-to-cell junctions, which in turn may illuminate the aetiology of some unsolved cases of epidermolysis bullosa. Further work will establish whether SOXF factors play a direct or an indirect role in regulating these genes. Our findings also provide the first evidence that SOXF factors could be involved in the pathophysiology of certain skin diseases, and open new perspectives to the development of novel therapeutic avenues.

## Materials and Methods

### Production of mice

All animal work was carried out following guidelines of the appropriate UK Home Office Project License (704793). *Ra^op^* mice were bred as described by Pennisi et al [16]. Embryo heads were fixed in 4% buffered paraformaldehyde, wax embedded, and serially sectioned at 7 µm. Sections were split over five to ten slides and prepared for histology, immunohistochemistry or radioactive *in situ* hybridization.

### Immunohistochemistry

After deparaffinization, sections were treated by proteinase K for antigen retrieval and then incubated with antibody to Collagen IV (Chemicon), integrin ß4 (Santa Cruz), integrin α6 (Abcam), plectin (Epitomics), nidogen (Santa Cruz), Keratin 5 (Gene Tex), BPAG1 (Santa Cruz) and von Willbrand factor (Abcam) after washing with PBS. As a negative control, normal rabbit serum or normal goat serum were used instead of primary antibody. To detect Collagen IV and integrin ß4, the sections were incubated with Cy3-conjugated secondary antibody (Jackson). To detect von Willbrand factor, three-step immunoperoxidase method employing avidin-biotin horseradish peroxidase (VECTOR) was performed. Tyramide signal amplification system (Parkin Elmer Life Science) was used for detecting Keratin 5, BPAG1, plectin and nidogen.

### *In Situ* Hybridisation

Radioactive section *in situ* hybridisation using ^35^S-UTP radiolabeled riboprobes was carried out according to Ohazama et al [51]. The radioactive antisense probes were generated from mouse *Claudin5* cDNA clones that were gifts from N. Sawada [52].

### RT-PCR

Epithelium was removed using Dispase in calcium- and magnesium-free PBS at 2 units per ml. E11.5 mandibles were incubated in Dispase solution for 10 minutes at 37°C. After incubation the mandibles were washed in PBS and the epithelium was dissected off using fine tungsten needles. Total RNA was isolated from the whole mandibles or epithelium by RNeasy (Qiagen). The RNA was converted into cDNA and was amplified for 30 cycles by Access RT-PCR System (Promega) using the following primers: *Lhx7*, 5′-CAAGGTGAATGACTTATGCTGGCA-3′ and 5′-GTCTTGCTCTGTGAGAAGGGCTC-3′; *Msx1*, 5′-TTCTCCAGCTCGCTCAGCCTCACC-3′ and 5′-TGCAGGACCGCCAAGAGGAAAAGAGAGGCC-3′; *Sox18*, 5′-TGCCACTACACTCCCCTACC-3′ and 5′-CCAGCTCTAAAGGCTGTTGC-3′; *Sox7*, 5′-GCCACGGCCACGTATTACAA-3′ and 5′-TGACCTCTTGCCACCAAGGA-3′; *Sox17*, 5′-AAGGCGAG GTGGTGGCGAGTAG-3′ and 5′-CCTGGCAGTCCCGATAGTGG-3′. Cycle parameters for amplification were as follows: *Lhx7*, 94°C for 30 s, 60°C for 45 s, and 68°C for 45 s; *Msx1*, 95°C for 30 s, 66°C for 30 s, and 72°C for 30 s; *Sox18*, 94°C for 30 s, 60°C for 45 s, and 68°C for 45 s; *Sox7*, 95°C for 30 s, 52°C for 45 s, and 72°C for 45 s; *Sox17*, 95°C for 30 s, 58°C for 45 s, and 72°C for 45 s.

### Quantitative-PCR

Total RNA was isolated from E14.5 embryo's whole heads using the RNeasy Mini Kit (Qiagen, Melbourne, Australia) and reverse-transcribed into cDNA with Superscript II Reverse Transcriptase according to manufacturer's instructions (Invitrogen, Melbourne, Australia). Real-time PCR analysis from each experimental sample was performed in a final volume of 20 ul with 25 pmol of each primer (Geneworks, Adelaide, Australia) and SYBR Green-1 (Applied Biosystems, Melbourne, Australia) using the Relative Standard Curve method on a ViiA 7 Real-time PCR System (Applied Biosystems, Melbourne, Australia). The PCR cycling conditions were: 95°C at 10 min for one cycle, then 40 cycles of amplification for 30 s at 95°C, 30 s at 60°C and 30 s at 72°C followed by a thermal melt profile for amplicon identification. Preparations of RNA template without reverse transcriptase were used as negative controls. C_t_ values were normalized to GAPDH [53]. Primers sequences for Plectin: 5′-tcacttcgcagagggaggt-3′ and 5′- gcacacggtctcgttcatc-3′ and Integrin-ß4 5′-cagcgtttctgatgacactga-3′ and 5′- tcattctgtgcagggagttg-3′.

### Ultrastructure analysis

Heads were fixed in 2.5% glutaraldehyde (phosphate buffer) overnight at 4°C and postfixed in 2% osmium tetroxide (Millonigs buffer) for 90 mins at 4°C after washing with phosphate buffer. Specimens were dehydrated through a graded series of ethanol and embedded in Epon 812-equivalent (TAAB Lab). Semi-thin sections (1 µm) were stained with toluidine blue for light microscopy analysis. Ultra-thin sections (40–90 nm) were cut, stained with uranyl acetate and lead citrate and examined with a Hitachi H7600 transmission electron microscope.

### Apoptotic activity

For detecting apoptoptic cells, we used the Apoptag plus fluorescein *in situ* apoptosis detection kit (Chemicon), according to manufacturer's protocol.

## Supporting Information

Figure S1 **The epithelial phenotype of heterozygous** ***Ra^op^*** **mice.** No detached epithelium was observed in heterozygous *Ra^op^* mice.
(TIF)
