# Supplementary figures and images for: Cytoplasmic Plaque Formation in Hemidesmosome Development Is Dependent on SoxF Transcription Factor Function
Source: PLoS One. 2012 Sep 4;7(9):e43857. doi: 10.1371/journal.pone.0043857 (PMC3433475; doi:10.1371/journal.pone.0043857)

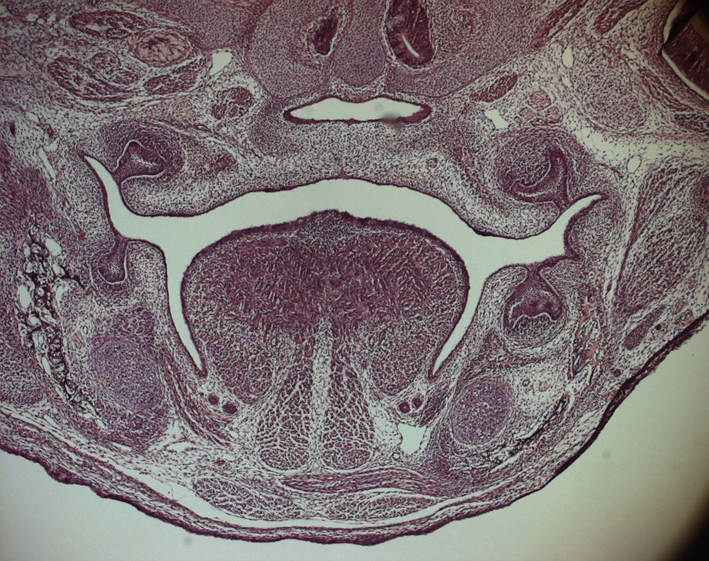

Supplement: Figure S1 — The epithelial phenotype of heterozygous Raop mice. No detached epithelium was observed in heterozygous Raop mice. (TIF) [file pone.0043857.s001.tif]
